# Switchable thulium-doped fiber laser from polarization rotation vector to scalar soliton

**DOI:** 10.1038/srep34844

**Published:** 2016-10-06

**Authors:** Zhichao Wu, Songnian Fu, Kai Jiang, Jue Song, Huizi Li, Ming Tang, Ping Shum, Deming Liu

**Affiliations:** 1 Wuhan National Laboratory for Optoelectronics, Huazhong University of Science and Technology, Wuhan, 430074, China; 2 National Engineering Laboratory of Next Generation Internet Access System, School of Optical and Electronic Information, Huazhong University of Science and Technology, Wuhan, 430074, China; 3 Centre for Optical Fibre Technology, Nanyang Technological University, Singapore, 637553, Singapore

## Abstract

We experimentally demonstrate switchable temporal soliton generation from a thulium-doped fiber laser (TDFL), using carbon nanotubes as the mode-locker. With the help of residual polarization dependent loss of a wavelength division multiplexer, a weak nonlinear polarization rotation (NPR) effect can be achieved within the laser cavity, which may provide joint contribution for passive mode-locking operation. By finely adjusting the polarization to alter the strength of NPR-based saturable absorption, the TDFL either approaches the operation regime of scalar soliton with strong NPR effect, or generates polarization rotation locked vector soliton (PRLVS) with weak NPR effect. The scalar solitons and PRLVSs possess 3-dB optical spectrum bandwidth of 2.2 nm and 2 nm, pulse-width of 1.8 ps and 2 ps, respectively. Moreover, the PRLVSs demonstrate a typical energy exchange between two polarized components on optical spectra and a period-doubling feature in time domain. Such operation principle can also be used in 1550 nm band fiber lasers and other nonlinear systems.

Passively mode-locked fiber lasers generating ultrashort and high-energy optical pulses have been widely studied over past decades, owing to its great advantages of high stability, simple structure and compact size^1^,^2^. Moreover, such lasers act as a convenient experimental platform for the investigation of nonlinear waves subject to periodic boundary conditions and easy modulation. To achieve passive mode-locking operation in a fiber laser, two main kinds of mode-locked techniques have been widely employed. The first approach is nonlinear switching based mode-locking, including nonlinear polarization rotation (NPR) and nonlinear amplifying loop mirror (NALM)^3^,^4^. Because these techniques either introduce polarization-dependent components or mechanism in the laser cavity, the output pulses always possess fixed linear polarization. Theoretically a scalar model is used to describe these output pulses, known as scalar solitons^5^. The other approach is material saturable absorber (SA) based mode-locking, as diverse as the semiconductor saturable absorber mirror (SESAM)^6^, graphene^7^, carbon nanotube (CNT)^8^, novel two-dimensional materials, such as MoS_2_^9^, and topological insulator^10^. Moreover, several previous researchers have investigated various fiber lasers mode-locked by CNT, such as multi-wavelength emission^11^ and tunable repetition rate^12^. The same as other SA materials, CNT with ideal polarization-independent feature can support vector solitons generation^13^.

Taking fiber birefringence into account, different types of vector solitons can be generated in fiber lasers. In general, a difference of group velocity between two orthogonal polarization components can cause them to separate temporally. However, this difference of group velocity can be compensated by a wavelength shift of two orthogonal components, and enable them to propagate as a single entity. Such type of soliton is known as group velocity locked vector soliton^14^,^15^. Apart from the group velocity, the phase velocities between two orthogonal components can also be locked as a result of the equilibrium among nonlinearity, dispersion, gain and loss, and form the so-called phase locked vector soliton^16^. Additionally, with the help of suitable cavity birefringence, both polarization locked vector soliton and polarization rotation locked vector soliton (PRLVS) can be observed in fiber lasers^17^,^18^. Various vector solitons, with attractive physical features, provide new perspectives and means to better understand the light polarization, which is a tradition but recently discovered very useful feature of laser physics^19^,^20^. Meanwhile, scalar solitons also play an important role in many theoretical researches and scientific applications, such as three-dimensional display^21^ and polarization division multiplexing transmission^22^. Therefore, it is worthwhile studying the polarization dynamics within laser cavity, as well as investigating the differences and correlation between scalar and vector solitons.

In previous studies of vector soliton, polarization dependence inside laser cavity has always been avoided if possible. All the physical SAs and optical components are assumed to be polarization-independent. In fact, some optical components, especially the wavelength division multiplexer (WDM) may possess a relatively large residual polarization dependent loss (PDL)^23^. This PDL gives us an inspiration to induce a controllable NPR effect in a SA-based mode-locked fiber laser. By adjusting the polarization state of light within the cavity, we are able to manipulate the strength of NPR-based saturable absorption and observe various polarization evolutions. Furthermore, researches on scalar and vector solitons so far have mainly concentrated upon 1550 nm wavelength region. Since the fiber birefringence comes from the deviations of the core shape from circularity, transverse internal stress or residual twist^15^, it is desired to observe similar soliton features of thulium-doped fiber lasers (TDFLs) operated at 2*μ*m region.

In this submission, we experimentally demonstrate switchable temporal soliton generation from a TDFL based on a combination of CNT and NPR effect induced by residual PDL arising in WDM. By finely adjusting the polarization controller, the fiber laser can generate either period-doubling PRLVSs or conventional scalar solitons. It is the first time that different polarization types of solitons have been generated and investigated within one laser cavity. This switchable operation of vector and scalar solitons may greatly enrich the understanding of soliton dynamics in laser cavities and find applications in a wide variety of optical fields.

## Experimental Results

**Figure 1:**
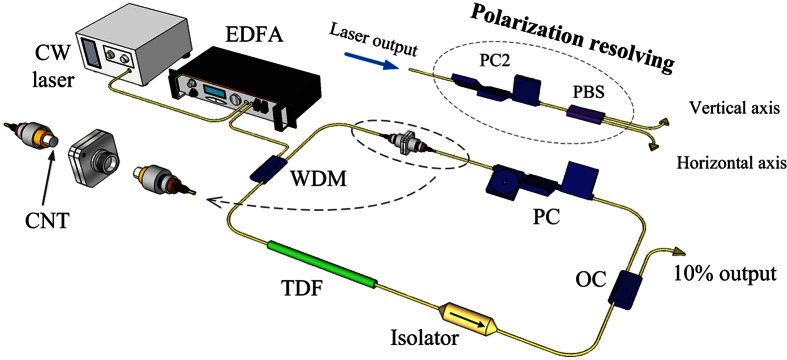
Schematic illustration of the experimental setup: the laser cavity and polarization resolved measurement. CW, continuous wave. EDFA: erbium-doped fiber amplifier. PC, polarization controller. OC, optical coupler. WDM, wavelength division multiplexer. TDF, thulium-doped fiber. PBS, polarization beam splitter.

The setup of the proposed fiber laser is schematically shown in Fig. 1. The CNT mode-locker is deposited on a standard FC/PC fiber end via the popular optically-driven deposition technique^24^,^25^. The CNTs are efficiently deposited in the region of the fiber core due to optimal interaction with radiation propagating in the fiber, while at the same time minimizing the waste of the CNTs during device preparation.

A continuous wave (CW) laser with 1 mW output power at 1570 nm is used as a seed source for an erbium-doped fiber amplifier (EDFA). The amplified signal with maximum output power of 5 W acts as a pump source for a 3.5-m commercial thulium-doped fiber (TDF, Nufern SM-TDF-10P/130-HE). The isolator is used to guarantee unidirectional propagation and to suppress detrimental reflections. The intra-cavity linear birefringence is adjusted by a polarization controller (PC). The laser output is achieved by a 10:90 optical coupler (OC). All the optical components within the cavity are fusion spliced, with a total cavity length of about 15 m. As for the polarization resolved measurement along the output port, a polarization maintaining fiber (PMF) pigtailed polarization beam splitter (PBS) is used to separate two orthogonal polarization components, and to simultaneously measure the polarization characteristics of generated solitons. In order to balance the fiber pigtail induced linear polarization rotation, we insert another PC between the laser output port and the PBS.

### Scalar soliton

**Figure 2:**
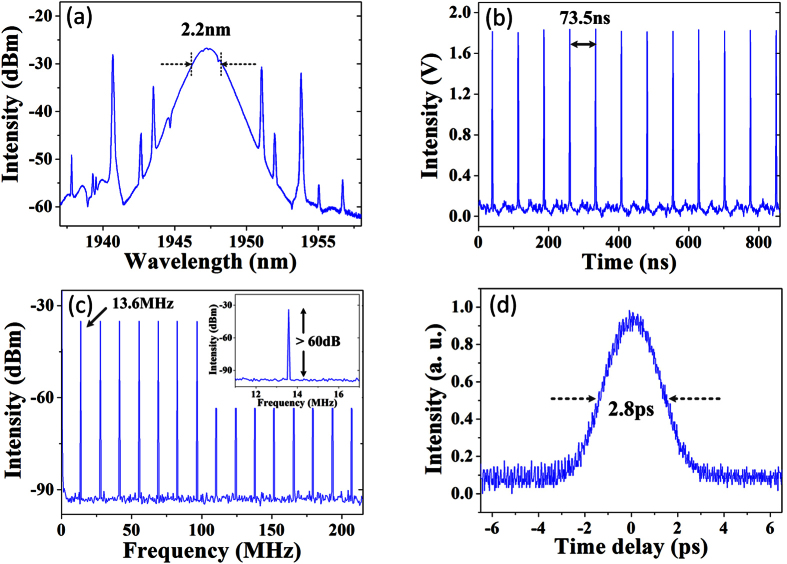
Scalar soliton state: (**a**) Optical spectra. (**b**) Oscilloscope trace of the pulse-train. (**c**) RF spectrum and fundamental frequency signal (inset). (**d**) autocorrelation trace.

Self-starting mode-locking at the central wavelength of 1947 nm can be observed, when the pump power is increased above 280 mW. We maintain the pump power to be 320 mW, and obtain a stable mode-locking operation with an output power of 4 mW, as shown in Fig. 2. Figure 2(a) presents a typical mode-locked spectrum with a 3-dB bandwidth of 2.2 nm. The sets of Kelly sidebands originating from spectral interference of dispersive waves are clearly observed, indicating that the fiber laser is operated under conventional soliton regime. The oscilloscope trace is shown in Fig. 2(b), where all pulses within the cavity possess exactly the same pulse height and the repetition rate of pulse-train is 13.6 MHz, which agrees well with the cavity length. The RF spectrum with a scanning range of 215 MHz is illustrated in Fig. 2(c). Obviously, there is no fluctuation in the broad spectrum, which confirms the stable mode-locking operation without Q-switching modulation. The smooth noise floor also demonstrates the fiber laser is operated with low amplitude noise. The inset shows the fundamental frequency with a resolution of 100 Hz. The signal-to-noise ratio (SNR) is about 60 dB. Figure 2(d) shows the pulse profile with the full-width of half maximum (FWHM) of 2.8 ps, corresponding to the pulse duration of 1.8 ps if a sech^2^ pulse profile shape is assumed. Therefore, the time-bandwidth product (TBP) of the pulses is ~0.32, indicating the pulse is almost transform-limited. For the case of femtosecond pulse generation in the TDFL, either more efficient pump scheme with the help of mode-locker with faster recovery time or chirp management along the TDFL output is desired.

**Figure 3:**
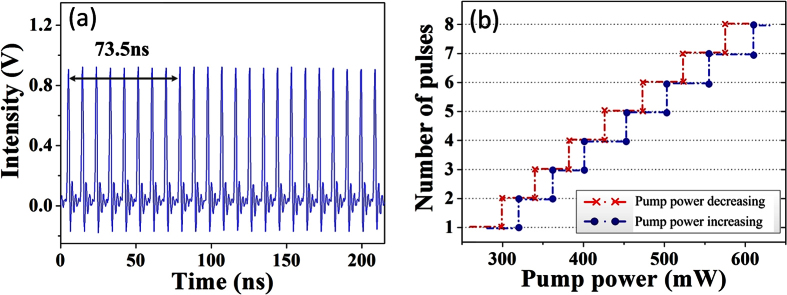
(**a**) 8th-order harmonic mode-locking at the pump power of 630 mW. (**b**) The number of pulses as a function of pump power.

**Figure 4:**
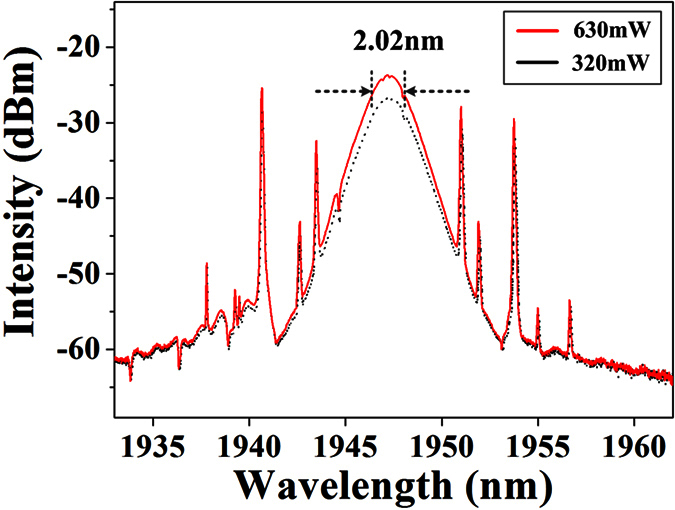
Optical spectra of scalar soliton at the pump power of 630 mW and 320 mW.

Next, we maintain polarization within the laser cavity and gradually increase the pump power to 630 mW. We can obtain a maximum of 8th-order harmonic mode-locking with a repetition rate of 108.8 MHz, as shown in Fig. 3(a). Then, we carefully decrease the pump power, the pulse-trains reduce one by one accordingly and the repetition frequency returns to fundamental mode-locking at the pump power of 270 mW. The formation and annihilation of each pulse-train verify the pumping parameter hysteresis^26^, as shown in Fig. 3(b). Especially, our mode-locking state always stably evolves with respect to the pump power adjustment. Furthermore, Fig. 4 shows the optical spectrum at the 630 mW pump power as a comparison with that at the 320 mW pump power. The 3-dB spectral bandwidth slightly reduces to 2.02 nm.

**Figure 5:**
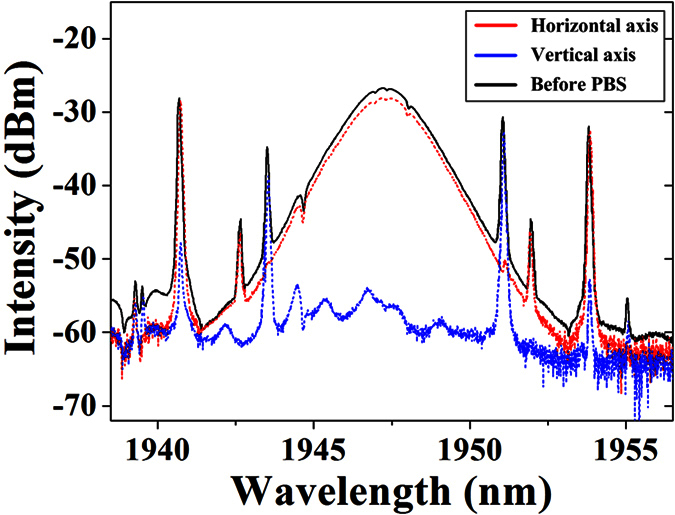
Optical spectra of the scalar soliton before and after polarization resolved measurement.

Then, to investigate the polarization features of generated solitons, we introduce the output of TDFL to the external polarization resolved configuration. We are able to simultaneously observe the optical spectra from two orthogonal polarization directions with the help of inline PBS. By finely adjusting PC2, the spectral intensity from two orthogonal directions always moves simultaneously with one rising up and the other falling down. In particular, we are able to achieve the maximum power of one direction while the other decreases to the lowest, as shown by the red and blue dash lines in Fig. 5. It is observed that the light at vertical axis is almost extinct, indicating that the soliton has linear polarization state along horizontal axis. This phenomenon is completely different from vector soliton case where two orthogonal components trap each other and always exist on both axes in fiber medium. In consequence, we can confirm the generation of scalar soliton on this scenario.

### Vector soliton

**Figure 6:**
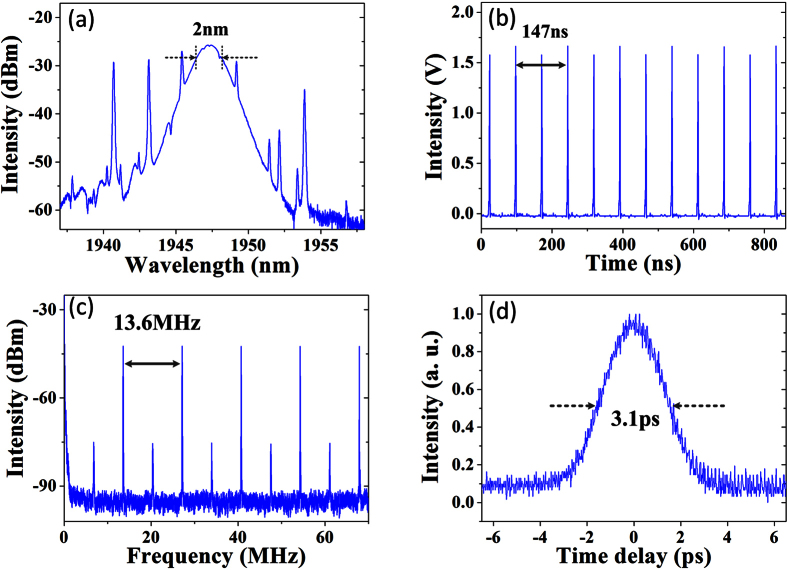
Period-doubling polarization rotation locked vector soliton state. (**a**) Optical spectra. (**b**) Oscilloscope trace of the pulse-train.(**c**) RF spectrum. (**d**) Autocorrelation trace.

If we keep the 320 mW pump power constant and further optimize the PC1, another stable mode-locking operation, which has typical features of period-doubling PRLVSs, can be obtained, as shown in Fig. 6. Figure 6(a) is the measured optical spectrum. The central wavelength stays unchanged at 1947 nm. However, the 3-dB bandwidth reduces to 2 nm, leading to an increase of the pulse profile width up to 3.1 ps, as shown in Fig. 6(d), which corresponds to the pulse duration of 2 ps if a sech^2^ pulse shape is assumed. Therefore, the output pulses maintain transform-limited characteristics in this case. Figure 6(b) illustrates the pulse-train with an evident period-doubling phenomenon characterized by the periodic intensity fluctuation between adjacent pulses. A basic feature of period-doubling pulses is a weak but obvious frequency component which appears at the position of the half cavity fundamental repetition rate, as shown by the RF spectrum with a scanning range of 70 MHz in Fig. 6(c).

**Figure 7:**
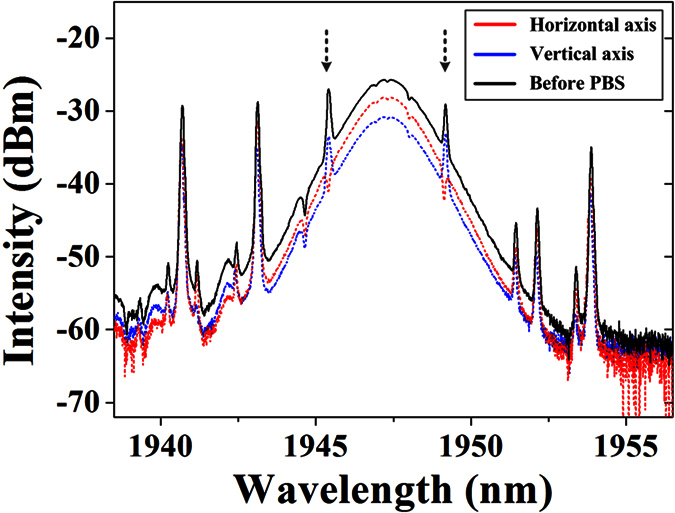
Optical spectra of the vector soliton before and after polarization resolved measurement.

**Figure 8:**
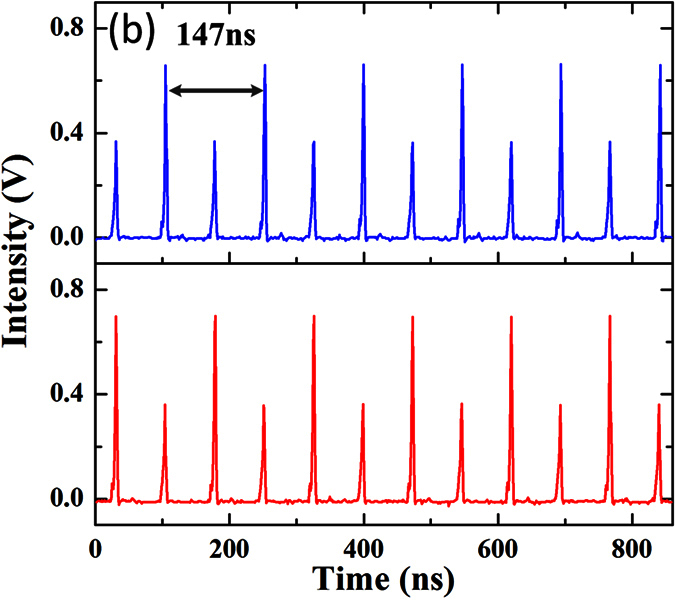
Oscilloscope traces of the vector soliton along horizontal axis (red) and vertical axis (blue).

**Figure 9:**
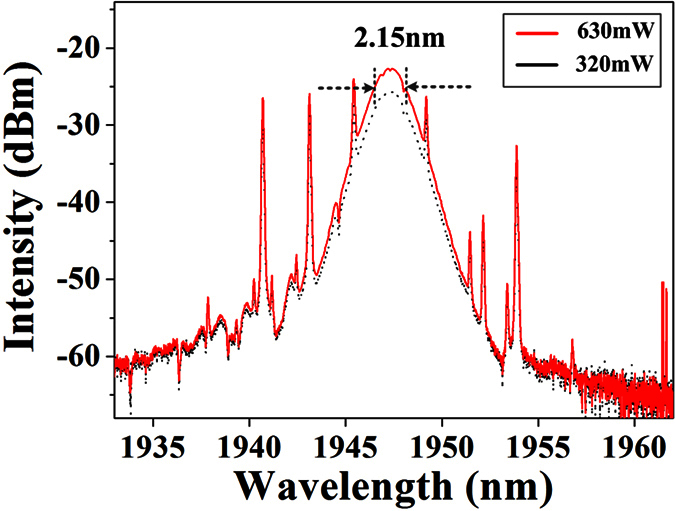
Optical spectra of vector soliton at the pump power of 630 mW and 320 mW.

To investigate the individual characteristics of two orthogonal components, we proceed with the same polarization resolved measurement. In this case, no matter how we adjust the PC2, we can no longer observe completed extinction at either orthogonal direction. Instead, the maximum intensity gap between the two axes is only ~3 dB, as shown in Fig. 7 with red and blue dash line. We note that, different from the optical spectrum of scalar soliton with only Kelly sidebands, there exists an extra pair of spectral sidebands for vector soliton, which are pointed by arrows in the Fig. 7. This pair of sidebands will shift their positions remarkably on the optical spectrum, when we carefully either adjust the PC inside laser cavity or alter the pump power, indicating that they are sensitive to the linear cavity birefringence. At the position of the extra sidebands after polarization resolved measurement, a peak is observed at the vertical component, while a dip is observed at the horizontal component. This peak-dip pair indicates that the pair of sidebands is generated due to the coherent energy exchange between two orthogonal polarization components^18^. We also simultaneously measure the temporal pulse-trains from two axes, as shown in Fig. 8. Compared to the original pulse-train in Fig. 6(b), we observe a larger periodic variation in the temporal pulse intensity at both axes. Obviously, the intensity of the vector solitons recurs every two cavity lengths, which leads to the period-doubling phenomenon. Further slightly adjusting the PC inside laser cavity, we can also get fundamental period state PRLVSs. In this case, the optical spectra demonstrate the same features as period-doubling PRLVSs with the extra sidebands, which confirms the coherent energy exchange between two polarization components of vector solitons. However, once the extra sidebands vanish from the optical spectrum, the mode-locking operation will turn back to the scalar soliton case, as shown in Fig. 2. Therefore, the existence of these extra sidebands can be treated as a switching symbol of generated soliton types. For the case of vector soliton, we also gradually increase the pump power, but harmonic mode-locking phenomenon is no longer observed. The pulse maintains fundamental frequency below 400 mW pump power and then turns into cluttered and irregular multipulses, instead of stable harmonic mode-locking state. At 630 mW pump power, the 3-dB spectral bandwidth becomes 2.15 nm, as shown in Fig. 9. Meanwhile, we observe that both the Kelly sidebands and energy exchange induced sidebands keep their position on the optical spectra.

## Numerical Simulation

To verify our experimental observations, we carry out the numerical simulations. The laser operation is simulated based on the coupled Ginzburg-Landau equations, which describe the pulse propagation. The pulse propagation in fibers is governed by:


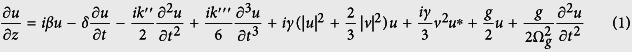



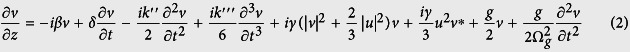


where u and v are normalized envelopes of the pulse along two orthogonal polarizations. 2*β* = 2*π*Δ*n*/*λ* is the wave number difference between two modes. *δ* = 2*βλ*/2*πc* is the inverse group velocity difference. *k*″ is the second-order dispersion coefficient, *k*″ is the third-order dispersion coefficient, and γ is the fiber nonlinearity coefficient. g and Ω_g_ represent the saturable gain coefficient and gain bandwidth of the TDF, respectively. We consider the gain saturation as:


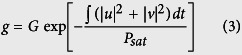


where *G* is the small signal gain coefficient and *P*_*sat*_ is the normalized saturation energy. The parameters are set as follows:


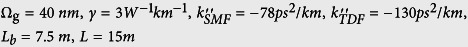


**Figure 10:**
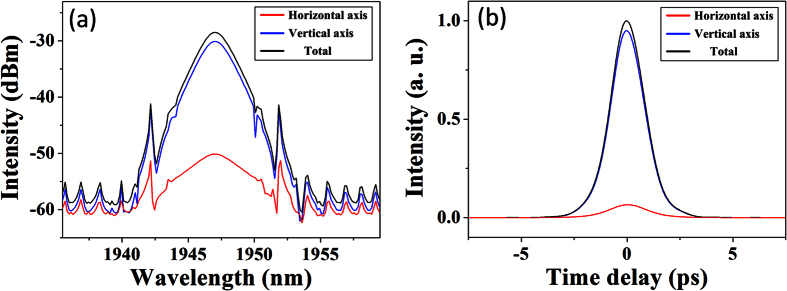
Numerical simulation of scalar soliton: (**a**) Optical spectra. (**b**) Pulse profiles.

**Figure 11:**
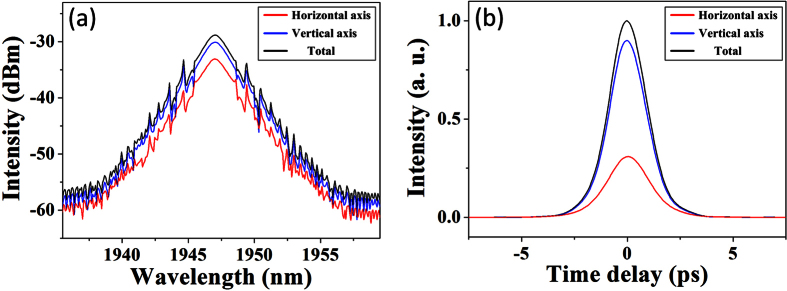
Numerical simulation of vector soliton: (**a**) Optical spectra. (**b**) Pulse profiles.

The performance of the CNT mode-locker is described by the rate equation^27^:


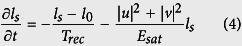


where *T*_*rec*_ is the absorption recovery time, *l*_0_ is the initial saturable absorption of the CNT. *E*_*sat*_ is the absorber saturation energy. We use the following parameters: *T*_*rec*_ = 6 ps, *E*_*sat*_ = 50 pJ, *l*_0_ = 0.12. The cavity transmission coefficient of the NPR operation is set as follows^28^:


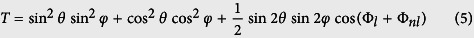


where *θ* is the angle between the fast axis of the equivalent polarization controller and the fast axis of the fiber; *φ* is the angle between the transmission axis of the WDM and the fast axis of the fiber. *ϕ*_*l*_ is the linear phase delay and *ϕ*_*nl*_ is the nonlinear phase delay. The linear phase delay includes the phase delay *ϕ*_*PC*_ induced by the PC, and the linear phase shift caused by the fiber birefringence. It is found that when *θ* = 0.125π, *φ* = 0.625π,*ϕ*_*PC*_ = 1.7π, the typical NPR based mode-locking can be obtained, as shown in Fig. 10. Figure 10(a) demonstrates the spectra from two orthogonal polarization directions. The light on vertical axis is nearly 20 dB greater than horizontal axis, corresponding to the scalar soliton state. Then, the intensity of NPR effect is theoretically manipulated by adjusting the linear cavity birefringence, which corresponds to the PC adjustment during experiment. When ϕ_PC_ is changed from 1.7π to 0.7π, the NPR effect becomes relatively weak, and CNT dominates the mode-locking operation. Figure 11 shows the CNT based mode-locking when ϕ_PC_ is set 0.7π and the NPR effect can be ignored. On this occasion, the lights of two polarization directions have only 3 dB spectral peak intensity difference, which corresponds to the vector soliton state. Figures 10(b) and 11(b) show the pulse profiles of scalar and vector soliton, respectively. The pulse widths of these two states are not the same, which is caused by the different recovery time and transmission coefficients of two different mode-locking regimes. The numerical simulation results agree well with our experimental observations.

## Discussion

Conventionally, in order to implement NPR technique in a ring fiber laser, a polarizer is inserted in the cavity, which forms a nonlinear birefringent filter in combination with the nonlinear fiber cavity. Under appropriate phase delay bias, the transmission of the nonlinear birefringent filter increases with the light intensity, leading to an artificial saturable absorption effect in the cavity^28^. However, a polarizer with polarization dependent loss (PDL) of ~30 dB always cause the restriction of light polarization, thus it is almost impossible to generate vector solitons in the cavity with NPR mode-locking mechanism. In terms of our laser cavity, we introduce a polarization sensitive component to realize the NPR mode-locking. After component characterization, the PDL of the used WDM is ~3 dB. Such residual PDL can also make the fiber laser self-start to mode lock. On the other hand, the PDL is not as strong as an inline fiber polarizer which completely restricts one polarization component. Consequently, it is possible to generate vector solitons in our laser cavity. In fact, the mode-locking operation is based on a combination of CNT mode-locker and NPR effect. Adjusting the PC inside cavity, we are able to alter the polarization state of light and change the strength of NPR-based saturable absorption. When the artificial saturable absorption is efficiently strong to dominate the mode-locking, the intrinsic polarization-selective property of NPR effect will restrain the pulse polarization and thus generate scalar soliton. On the other hand, when the artificial saturable absorption is relatively weak and CNT mode-locker makes more contribution to mode-locking operation, the fiber laser can be operated at vector soliton state. To further verify the mechanism, we separately decrease the pump power from the two mode-locking operations. The scalar solitons degenerate to CW laser emission, when the pump power is reduced below 270 mW. While the vector solitons can be maintained till 220 mW. The obvious pump power difference can be explained by the different energy requirement for the NPR and SA-based mode-locking.

## Conclusions

In conclusion, we experimentally demonstrate switchable solitons generation from a TDFL based on the CNT. By finely adjusting the polarization to alter the strength of NPR-based saturable absorption, the fiber laser can be operated at either PRLVSs or scalar solitons. After polarization solved measurement at the output port of TDFL, the scalar solitons are characterized by complete extinction in one polarization direction, while the PRLVSs present a typical period-doubling mode-locking state. This switchable working regime is novel and likely to be applicable to a variety of other nonlinear systems.

## Methods

An optical spectrum analyzer (OSA, Yokogawa AQ6375) with a resolution of 0.05 nm is used to observe the optical spectra. Meanwhile, a real-time oscilloscope (OSC, Agilent 54641A) with a bandwidth of 350 MHz is used to monitor the temporal pulses with the help of a photodetector (PD, EOT ET-5000F) of 12.5 GHz. Moreover, the radio-frequency (RF) spectrum is characterized by a signal source analyzer (R&S FSUP). Finally, the pulse profile is measured by a commercial autocorrelator (FR-103XL).

## Additional Information

**How to cite this article**: Wu, Z. *et al.* Switchable thulium-doped fiber laser from polarization rotation vector to scalar soliton. *Sci. Rep.* **6**, 34844; doi: 10.1038/srep34844 (2016).
